# Single-cell transcriptomics dissects the transcriptome alterations of hematopoietic stem cells in myelodysplastic neoplasms

**DOI:** 10.1186/s12967-024-05165-z

**Published:** 2024-04-17

**Authors:** Xiangzong Zeng, Yichen Wang, Min Dai, Wei Li, Qingtian Huang, Lingsha Qin, Yuquan Li, Yanwen Yan, Xiangjun Xue, Fang Yi, Wenhao Li, Langyu He, Qifa Liu, Ling Qi

**Affiliations:** 1 Department of Hematology, Affiliated Qingyuan Hospital, Guangzhou Medical University, Qingyuan People’s Hospital, Qingyuan, 511518 China; 2 Division of Gastroenterology, Institute of Digestive Disease, Affiliated Qingyuan Hospital, Guangzhou Medical University, Qingyuan People’s Hospital, Qingyuan, 511518 China; 3 Department of Hematology, Nanfang Hospital, Southern Medical University, Guangzhou, 510515 China; 4 Department of Blood Transfusion, Affiliated Qingyuan Hospital, Guangzhou Medical University, Qingyuan People’s Hospital, Qingyuan, 511518 China

**Keywords:** Myelodysplastic neoplasms, Transcriptome alterations, Hematopoietic stem cells, Leukemic transformation

## Abstract

**Background:**

Myelodysplastic neoplasms (MDS) are myeloid neoplasms characterized by disordered differentiation of hematopoietic stem cells and a predisposition to acute myeloid leukemia (AML). The underline pathogenesis remains unclear.

**Methods:**

In this study, the trajectory of differentiation and mechanisms of leukemic transformation were explored through bioinformatics analysis of single-cell RNA-Seq data from hematopoietic stem and progenitor cells (HSPCs) in MDS patients.

**Results:**

Among the HSPC clusters, the proportion of common myeloid progenitor (CMP) was the main cell cluster in the patients with excess blasts (EB)/ secondary AML. Cell cycle analysis indicated the CMP of MDS patients were in an active proliferative state. The genes involved in the cell proliferation, such as MAML3 and PLCB1, were up-regulated in MDS CMP. Further validation analysis indicated that the expression levels of MAML3 and PLCB1 in patients with MDS-EB were significantly higher than those without EB. Patients with high expression of PLCB1 had a higher risk of transformation to AML. PLCB1 inhibitor can suppress proliferation, induce cell cycle arrest, and activate apoptosis of leukemic cells in vitro.

**Conclusion:**

This study revealed the transcriptomic change of HSPCs in MDS patients along the pseudotime and indicated that PLCB1 plays a key role in the transformation of MDS into leukemia.

**Supplementary Information:**

The online version contains supplementary material available at 10.1186/s12967-024-05165-z.

## Introduction

Myelodysplastic neoplasms (MDS) are a group of heterogeneous malignant hematopoietic stem cell (HSC) diseases which are characterized by disordered growth and differentiation of hematopoietic stem cells and a high risk of transformation to acute myeloid leukemia (AML) [1]. Although multiple new drugs are currently available, effective treatment options remain limited for MDS [2]. The current standard care is still hypomethylating agent (HMA)-based therapy. However, although HMAs have resulted in clinical responses in about 50% MDS patients, the disease eventually becomes resistant to these agents and progresses to secondary AML (sAML) [3, 4]. MDS is a disease of stem cells, and MDS stem cells are functionally critical for the initiation, transformation, and relapse of disease [5, 6]. Therefore, therapies targeting stem cells are promising future curative strategies in MDS.

Some studies revealed that there are quantitative and qualitative alterations in hematopoietic stem and progenitor cells (HSPCs) from MDS patients [7–9]. Several cell surface markers including interleukin-1 (IL-1) receptor accessory protein (IL1RAP), T-cell immunoglobulin mucin-3 (TIM3), CD123, CD47, CD99 and so on, have been identified to differentiate MDS HSPCs from healthy counterparts [8, 10–13]. In addition, MDS HSC are dysfunction with dysregulated gene regulation, increased inflammatory signaling, alterations in RNA splicing and ribosome assembly/translation [14]. However, even though there is increasing studies for the alterations of MDS HSC and a number of targets have been demonstrated as potential therapeutic targets in MDS, the ideal therapies in MDS which target the malignant HSCs have not been found. An improved understanding of the molecular pathways that regulate these disease-initiating stem cells is still required for the development of future targeted therapies.

The MDS is highly heterogeneous in clinic and may be mild and stable for many years or may progress rapidly to AML [15]. The underlying pathogenesis remains unclear. Recently, the single-cell RNA sequencing (scRNA-seq) technology provides an unprecedented opportunity to deepened our understanding of normal hematopoiesis by identifying the transitional cell states between classical hematopoietic hierarchy stages [16, 17]. In this study, we revealed the overall transcriptome alterations in HSPCs between MDS patients and HCs along the pseudotime trajectory using scRNA-seq data. We found the major amplifying population in HSPCs in MDS with excess blasts (EB) (MDS-EB)/secondary AML is CMP and the high expression of PLCB1 might play an important role in the transformation of MDS into leukemia.

## Results

### Single-cell RNA-Seq reveals the alteration of HSPC populations in MDS

**Fig. 1:**
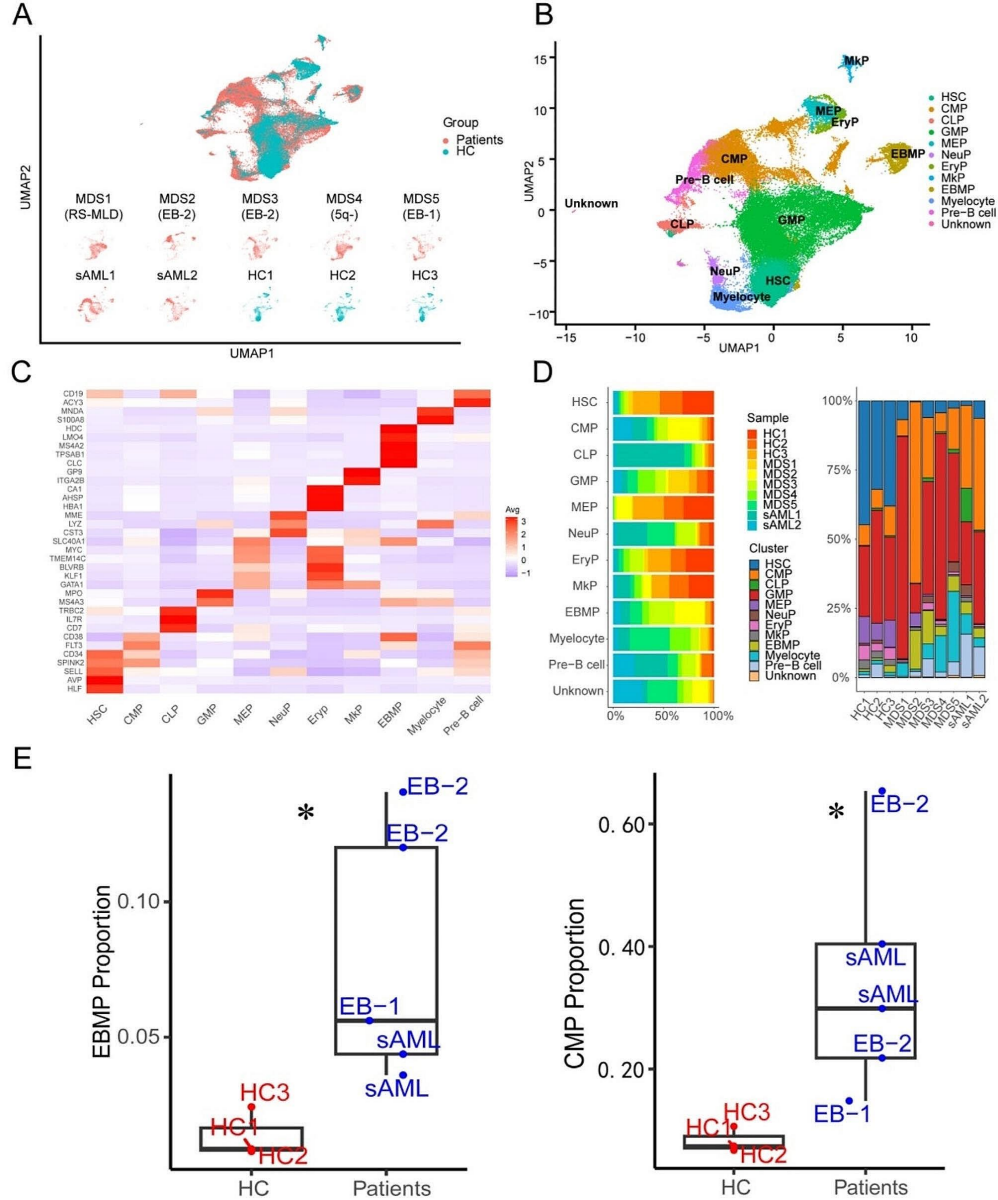
Identifying HSPC populations in BM. (**A**) UMAP of human BM cells from 7 MDS/sAML patients and 3 healthy controls. *RS-MLD*, ring sideroblasts-multilineage dysplasia; *EB-2*, excess blasts-2; *5q-*, del(5q); *EB-1*, excess blasts-1; *sAML*, secondary acute myeloid leukemia; *HC*, healthy control. Cells are color-coded according to samples. (**B**) UMAP of HSPC subclusters. Cells are color-coded according to the defined subset. (**C**) Heatmap displaying scaled expression of canonical cell type-associated genes for clusters. (**D**) Stacked bar plots show the percentage of sample contributions per annotated cell type (left) and the percentage of annotated cell type contributions per sample (right). (**E**) Boxplot showing the fraction of CMP and EBMP cluster in HCs (red) and MDS-EB/sAML patients (blue) samples. The *p* values were calculated using two-tailed Student’s t test; *p < 0.05

All the scRNA-seq datas came from public databases. The scRNA-seq data of lineage negative (Lin−) bone marrow (BM) cells from 7 patients with MDS [18] (2 of which has been had progressed to sAML), and 3 healthy donors [19] was performed uniform manifold approximation and projection (UMAP) analysis (Fig. 1A). The detailed UMAP of HSPCs are shown in supplemental Fig. S1A-D. In order to be consistent with the description in the article which provides the scRNA-seq data, the disease classification of patients was still based on the 2016 revision of the World Health Organization classification of myeloid neoplasms [20]. These 50,348 BM-derived cells segregated into 11 populations with distinct gene expression patterns (Fig. 1B-C). The expression of canonical marker genes during hematopoietic development are shown in supplemental Fig. S1E. These populations included HSC, CMP, granulocyte monocyte progenitor (GMP), common lymphoid progenitors (CLP), megakaryocyte-erythroid progenitors (MEP), neutrophil progenitors (NeuP), erythroid progenitors (EryP), megakaryocytic progenitors (MkP), eosinophil-basophil-mast-cell progenitors (EBMP), myelocytes, pre-B cell populations (pre-B) and one unknown cluster (could not be identified). Among the HSPC clusters, the major components were CMP and GMP in MDS/sAML and the proportion of CMP was the main cell cluster in the patients with excess blasts (EB)/sAML(supplemental Fig. S1F, Fig. 1D-E). The proportion of HSC, MEP, Eryp and MkP were significantly lower in MDS/sAML patients than in HCs (supplemental Fig. S1F), suggesting a differentiation block in the transition from CMP to MEP, which were in line with previous reports [7, 8].

### Pseudotime analysis reveals the abnormal trajectories of HSPCs differentiation in MDS

**Fig. 2:**
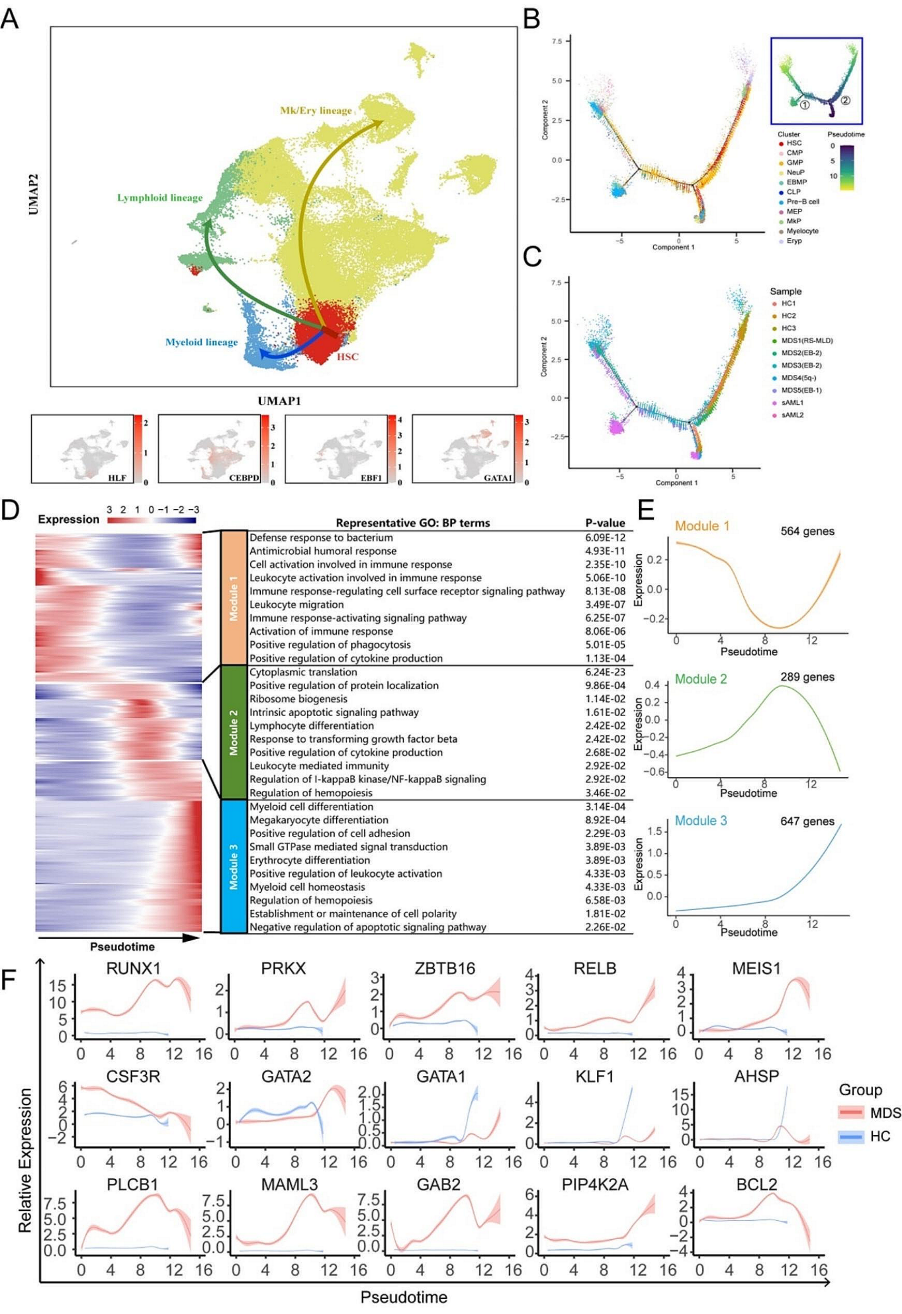
Trajectory inference of the hematopoietic lineages and dynamic gene expression patterns of HSPCs. (**A**) UMAP of human BM cells. Arrows indicate main directions of differentiation, inferred from analysis of typical marker genes. (**B**) Reconstructed principal component graph of cell differentiation trajectory of HSPCs, colored by subpopulation identities. The upper-right trajectory plot in the square indicates the direction of pseudotime. (**C**) Pseudotime-ordered analysis of HSPCs from the MDS/sAML and HC samples. (**D**) Heatmap of gene expression dynamic trends for genes along the pseudotime in MDS/sAML patients and HCs (cataloged hierarchically into three gene modules). *P* value < 0.05 was considered statistically significant for Gene Ontology (GO) enrichment analysis. (**E**) Loess-smoothed curves fitted to the z scored averaged expression of genes in modules 1–3 along the pseudotime trajectory. (**F**) Two-dimensional plots showing the dynamic expression of significantly enhanced genes in MDS/sAML patients compared with HCs along the pseudotime in module 3.|LogFC|≥1 and *p* value < 0.05 were used to define differentially expressed genes (DEGs)

To identify the dynamic gene expressions along differentiation trajectories, trajectory analysis was performed using Monocle 2 [21]. There were three main directions of differentiation that resemble the main hematopoietic lineages: the myeloid, megakaryocytic/erythroid, and lymphoid lineage, inferred from analysis of typical marker genes (CEBPD, GATA1 and EBF1) [22–24](Fig. 2A). Distinct differentiation trajectories were observed during hematopoiesis between MDS patients and HCs (Fig. 2B-C). The distribution of HSPC types along the pseudotime are shown in supplemental Fig. S2A. According to the transcriptional changes associated with transitional states, we identified three different gene expression modules (modules 1–3) (Fig. 2D-E). Analysis of Gene Ontology (GO) showed that genes of module 1 were enriched in these biological process (BP) terms: defense response to bacterium, antimicrobial humoral response, cell activation involved in immune response, leukocyte activation involved in immune response and immune response-regulating cell surface receptor signaling pathway. Genes of module 2 were enriched in cytoplasmic translation, positive regulation of protein localization, ribosome biogenesis, intrinsic apoptotic signaling pathway and lymphocyte differentiation. Genes of module 3 were enriched in myeloid cell differentiation, megakaryocyte differentiation, positive regulation of cell adhesion, small GTPase mediated signal transduction and erythrocyte differentiation. The dynamic expression of scores for representative pathway in module 1–3 are shown in supplemental Fig. S2B-D. The direction of pseudotime and DEGs of different branches (different cell fates in branch 2) were shown in supplemental Fig. S3A-B. The top GO BP pathways of different clusters were listed in supplemental Fig. S3C.

Next, the enhanced genes along the pseudotime were compared between MDS patients and HCs. In module 1, DEFA3, S100A8 and S100A9, which involved in multiple inflammatory pathways were the top 3 up-regulated DEGs along the pseudotime. The dynamic expression of representative immune-related genes are shown in supplemental Fig. S2E. In module 2, the expressions of genes involved in ribosome biogenesis (such as RPL5, RPL14, RPL21, RPL10 and NPM1, etc.) were lower in MDS/sAML (supplemental Fig. S2F). In module 3, the results indicated that RUNX1 [25, 26], ZBTB16 [27], GATA2 [28, 29] and MEIS1 [30] which involved in the myeloid cell differentiation and the pathogenesis of acute leukemia showed higher levels of expression in cells from the MDS across differentiation (Fig. 2F). Moreover, the expression of genes involved in macrophage and granulocyte differentiation (PRKX [31], RELB [32] and CSF3R [33]), cell proliferation (PIP4K2A [34]) and anti-apoptotic regulator B cell lymphoma 2 (BCL2 [35]) were also higher in MDS/sAML (Fig. 2F). However, genes involved in erythrocyte differentiation (GATA1 [36] and KLF1 [37]) and hemoglobin assembly and stability (AHSP [38]) showed lower levels of expression in MDS/sAML patients compared to HCs (Fig. 2F).

### Transcriptional changes of HSPCs in MDS

**Fig. 3:**
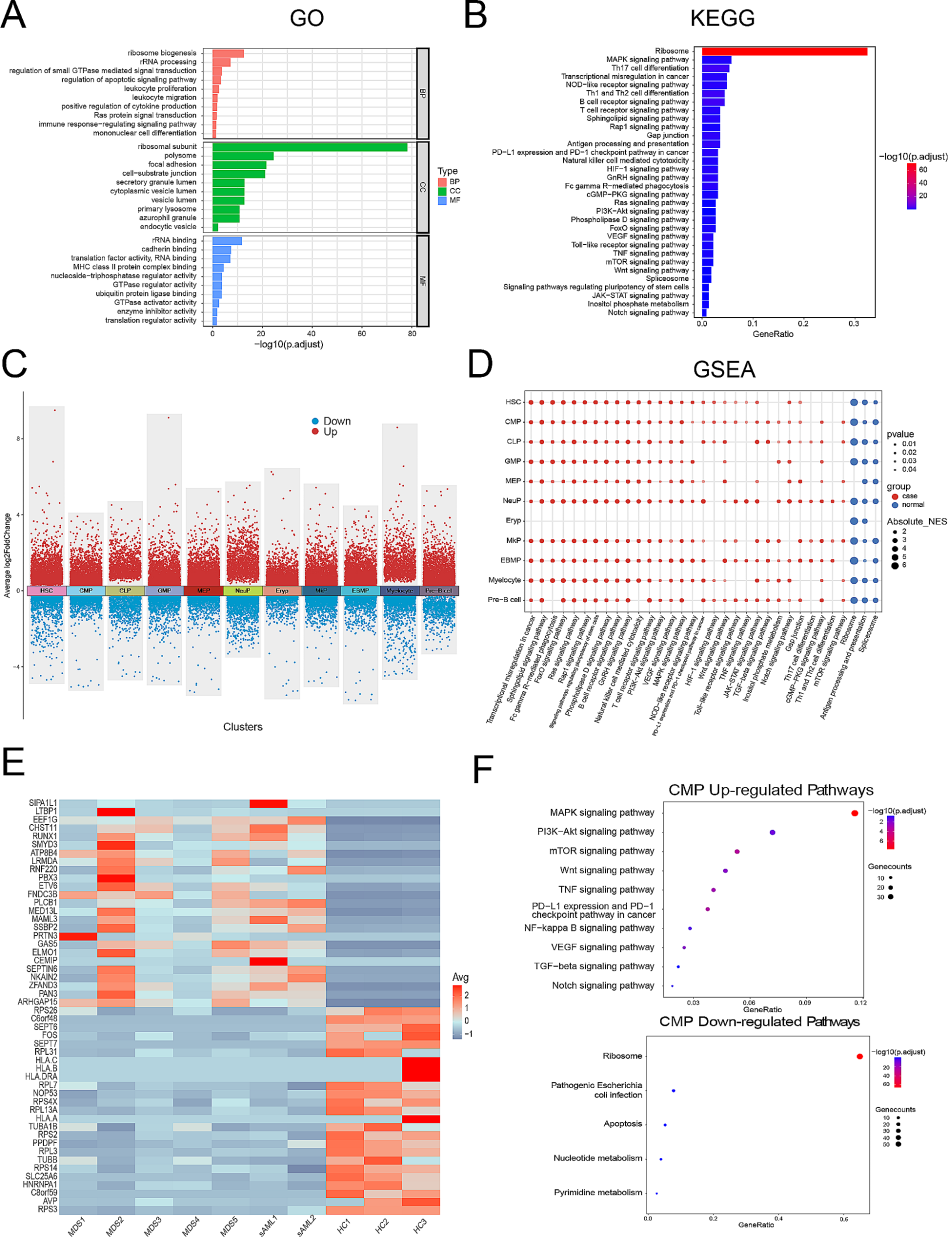
Transcriptional changes of HSPCs in MDS. (**A**) Functional enrichment bar chart (GO terms) of DEGs in total HSPCs of MDS/sAML patients compared with HCs.|LogFC|≥1, adjusted *p* value < 0.05 were used to define DEGs. Adjusted *p* value < 0.05 was considered statistically significant for GO enrichment analysis. (**B**) Functional enrichment bar chart (KEGG terms) of DEGs in total HSPCs of MDS/sAML patients compared with HCs. Adjusted *p* value < 0.05 was considered statistically significant for KEGG enrichment analysis. (**C**) DEGs analysis showing up-(red) and down-(blue) regulated genes in MDS/sAML across the 11 HSPC clusters. Adjusted *p* value < 0.05 were used to define DEGs. (**D**) Dot plot of enriched terms after performing gene set enrichment analysis (GSEA) for each identified cluster. Dot color represents the enriched group, size indicates the NES absolute value, and transparency indicates the *p*-value. (**E**) Heatmap of top 60 differentially expressed genes in CMP of MDS/sAML patients compared with HCs.|LogFC|≥1 and adjusted *p* value < 0.05 were used to define DEGs. (**F**) Dot plot of enriched upregulated and downregulated KEGG terms in CMP of MDS/sAML patients. Adjusted *p* value < 0.05 was considered statistically significant for KEGG enrichment analysis

We assessed transcriptome alterations in MDS/sAML HSPCs patients compared with HCs, yielding a total of 940 differentially expressed genes (DEGs), with 765 up-regulated genes and 175 down-regulated genes (|Log fold change (LogFC)|≥1, adjusted *p* value < 0.05). The top 200 differentially expressed genes were shown in heatmap (supplemental Fig. S4A). Through functional enrichment analysis, including GO and KEGG pathway analysis, we found these DEGs were mainly involved in ribosome biogenesis, RNA metabolism, regulation of apoptotic signaling pathway, leukocyte proliferation and immune dysfunction (Fig. 3A-B). The transcriptional changes in total HSPCs of 5 MDS patients compared with 2 sAML patients were shown in supplemental Fig. S4B-D. The functional enrichment analysis showed DEGs were mainly involved in immune dysfunction (such as antigen receptor − mediated signaling pathway, immune response − regulating cell surface receptor signaling pathway, antigen processing and presentation of exogenous peptide antigen via MHC class II, T cell differentiation, etc.) and signal transduction pathways (such as Phospholipase D signaling pathway, Sphingolipid signaling pathway, cGMP − PKG signaling pathway, etc.) (supplemental Fig. S4C).

Next, transcriptome alterations were assessed in each HSPC subset of MDS (Fig. 3C), and the gene set enrichment analysis (GSEA) was performed for each identified cluster (Fig. 3D). The results indicated that most HSPC subsets of MDS patients exhibited a generalized enrichment of inflammation, signal transduction, or hypoxia pathways, while ribosome, antigen processing and presentation and spliceosome pathway were enriched in HCs (Fig. 3D).

As mentioned above, CMP was the main cell cluster in the patients with MDS-EB/sAML. We next delineated the transcriptional changes of CMP in MDS (Fig. 3E). In particular, genes associated with hematopoiesis (RUNX1, ETV6), cell proliferation (RNF220, PLCB1 and MAML3) or DNA damage response (SSBP2) were upregulated in MDS. On the contrast, genes associated with ribosome biogenesis (RPS26, RPL31, RPL7, etc.), major histocompatibility complex (HLA.C, HLA.B, HLA.DRA, etc.) or apoptosis (SLC25A6) were downregulated in MDS. KEGG enrichment analysis showed that up-regulated DEGs in CMP of MDS patients enriched in cell growth, development, differentiation and inflammation pathways, such as MAPK signaling pathway, PI3K-Akt signaling pathway, mTOR signaling pathway and so on (Fig. 3F). On the other hand, down-regulated DEGs enriched in ribosome, pathogenic Escherichia cell infection, apoptosis, etc. (Fig. 3F)

### MAML3 and PLCB1 were upregulated in patients with MDS-EB

**Fig. 4:**
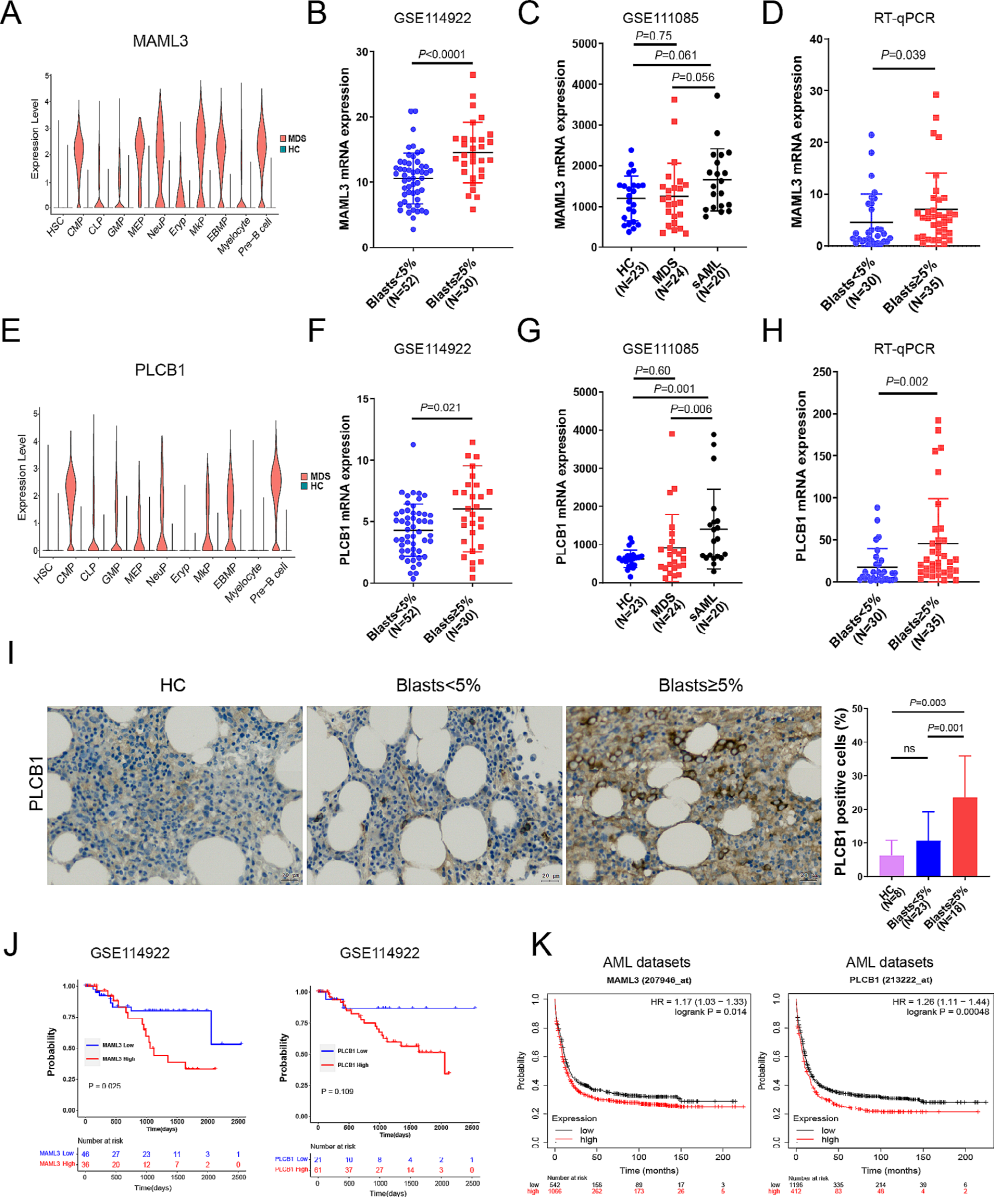
Further investigation of MAML3 and PLCB1 in MDS. (**A**) Violin plots showing the expression of MAML3 in each cell cluster. (**B**, **C**) Identification of MAML3 as an upregulated gene in CD34 + HSPCs by integrating multiple Gene Expression Omnibus (GEO) datasets (GSE114922, and GSE111085). (**D**) RT-qPCR analyses of MAML3 mRNA in CD34 + HSPCs of MDS patients. (**E**) Violin plots showing the expression of PLCB1 in each cell cluster. (**F**, **G**) Identification of PLCB1 as an upregulated gene in CD34 + HSPCs by integrating GEO datasets (GSE114922 and GSE111085). (H) RT-qPCR analyses of PLCB1 mRNA in CD34 + HSPCs of MDS patients. (**I**) Left, representative images of immunohistochemical (IHC) staining of PLCB1 in BM biopsy specimens. Scale bar, 20 μm; Right, statistical analysis of PLCB1 immunohistochemistry score. (**J**) Kaplan–Meier survival analysis of overall survival (OS) in MDS patients. (**K**) OS of AML patients calculated by the online survival analysis tool “Kaplan-Meier Plotter” (KM plotter) database, including GSE1159, GSE12417, GSE37642, GSE6891 and GSE 8970 datasets

Based on the proportion of HSPC subsets, cell trajectories, differential genes and functional enrichment, we hypothesized that the proliferation of CMP may be an important event in leukemic transformation. Therefore, we focused on the up-regulated DEGs in CMP which involved in the cell proliferation. MAML3 is a transcriptional co-activator of Notch signaling pathway, contributing to cancer cells proliferation in acute lymphoblastic leukemia [39] and some solid tumors [40, 41]. PLCB1, is a kind of phospholipase C, involving in the phosphoinositide-dependent signal transduction pathway and Wnt signaling pathway. It has been implicated in cell growth and proliferation, cell cycle regulation and cellular differentiation [42]. Thus, MAML3 and PLCB1 were selected for further verification. The expression of these two genes in HSPC subsets are shown in Fig. 4A and E.

Firstly, we analyzed the transcriptome sequencing data of HSPCs (CD34+) from GSE114922 and GSE111085 datasets. The results of GSE114922 indicated that the expression levels of MAML3 and PLCB1 in patients with MDS-EB were significantly higher than those without EB (*p* < 0.001, *p* = 0.021, respectively) (Fig. 4B and F). The other dataset GSE111085 revealed that patients whose illness transformed to AML within 12 months (sAML) had higher expression levels of PLCB1 than those whose disease remained stable MDS (*p* = 0.006) (Fig. 4G). In addition, an increasing trend towards the expression levels of MAML3 was observed in sAML (*p* = 0.056) (Fig. 4C). There was no significant difference in the expression of MAML3 and PLCB1 between MDS patients and HCs (both *p* > 0.05) (Fig. 4C and G). Secondly, we collected BM samples from 65 MDS patients. Reverse Transcription-Quantitative Polymerase Chain Reaction (RT-qPCR) was used to detect the expression of MAML3 and PLCB1 in BM CD34 + cells. The results also showed that the expression levels of MAML3 and PLCB1 in patients with MDS-EB were significantly higher than those without EB (*p* = 0.039, *p* = 0.002, respectively) (Fig. 4D and H). Based on the median mRNA expression of PLCB1, we divided the patients into 2 groups: PLCB1-Low and PLCB1-High groups. The numbers of white blood cell count and neutrophils at diagnosis were lower and the proportion of patients with “very-high” risk was higher in the PLCB1-High group compared to the PLCB1-Low group (*p* = 0.042, *p* = 0.025, *p* = 0.007, respectively) (supplemental Table S1). Moreover, the proportion of patients who transformed into AML was also higher in the PLCB1-High group (*p* = 0.019) (supplemental Table S1). In terms of protein expression, the expression of PLCB1 in bone marrow biopsy specimens of MDS patients was detected by immunohistochemical method, and it was found that the expression of PLCB1 in patients with MDS-EB was significantly higher than that in HCs and those without EB (*p* = 0.003, *p* = 0.001, respectively) (Fig. 4I).

Finally, the patients were divided into high and low expression groups according to the best cut-off value of mRNA expression from GSE114922 dataset. Survival analysis of this dataset indicated MDS patients with high expression of MAML3 had shorter overall survival (OS) (*p* = 0.025) (Fig. 4J). A downward trend for OS was also observed in patients with high expression of PLCB1 (*p* = 0.109) (Fig. 4J). Moreover, we found that OS was significantly lower among AML patients with high expression of MAML3 or PLCB1, calculated by the online survival analysis tool “Kaplan-Meier Plotter” (KM plotter) database, including GSE1159, GSE12417, GSE37642, GSE6891 and GSE8970 datasets (Fig. 4K).

### PLCB1 inhibitor U73122 suppress proliferation, induce cell cycle arrest, and activate apoptosis of leukemic cells in vitro

**Fig. 5:**
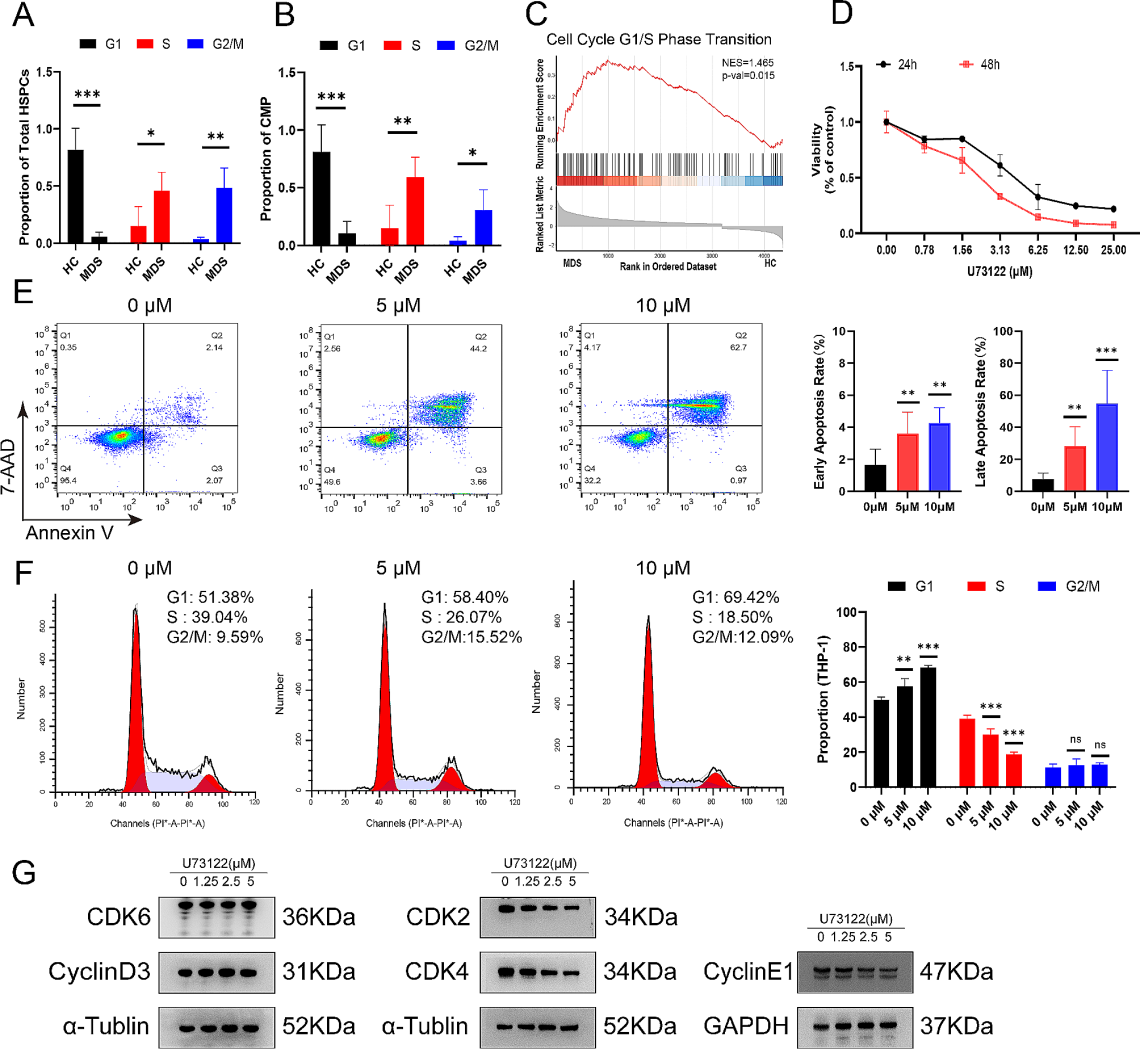
PLCB1 inhibitor U73122 suppress proliferation, induce cell cycle arrest, and activate apoptosis of leukemic cells in vitro. (**A**) Sample-wise proportion of cells in G1, S and G2M phase in total HSPCs. (**B**) Sample-wise proportion of cells in G1, S and G2M phase in CMP. (**C**) GSEA plots showing pathways of cell cycle G1/S phase transition enriched in CMP of MDS patients. (**D**) CCK-8 assay of cell viability in THP-1 cell line with different concentrations of U73122 for 24 and 48 h respectively. (**E**) Cell apoptosis analysis of THP-1 cells treated with 0µM, 5µM or 10µM U73122 for 24 h. (**F**) Cell cycle analysis of THP-1 cells treated with 0µM, 5µM or 10µM U73122 for 24 h. (**G**) CDK6 and cyclin D3 expression stained unchanged while CDK2, CDK4 and cyclin E1 expression were down-regulated in THP-1 cells after U73122 treatment

PLCB1 is implicated in cell proliferation and differentiation, and it plays an important role in cell cycle, especially G1/S transition [43]. Thus, we used cyclone tool of R package scran [44] to calculate the cell cycle scores of HSPCs. The results indicated that the proportion of total HSPCs in G1 phase of MDS patients was significantly lower, while the proportion of cells in S and G2/M phase were higher when compared to those of HCs (Fig. 5A). Similar results were observed in CMP subset (Fig. 5B). GSEA functional enrichment showed cell cycle G1/S phase transition pathway was enriched in CMP of MDS patients compared to HCs (Fig. 5C). These results indicated CMP of MDS patients were in an active proliferative state.

**Fig. 6:**
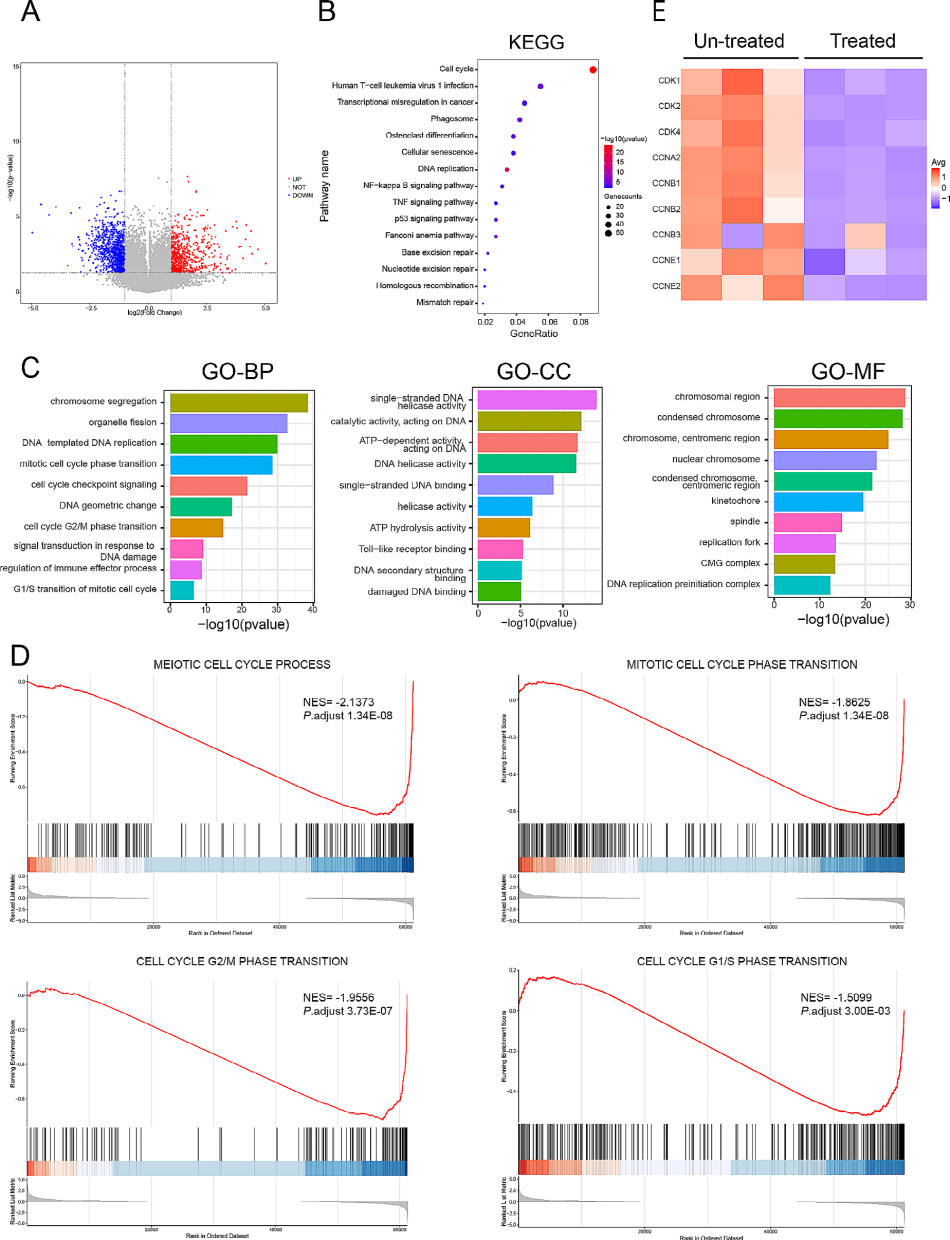
PLCB1 inhibitor U73122 affects cell cycle-related signaling pathways. (**A**) Volcano plots showing 1646 genes with the most statistically significant differences between U73122 (5µM) treated and untreated THP-1 cells.|LogFC|≥1 and *p* value < 0.05 were used to define DEGs. (**B**) Functional enrichment bar chart (KEGG terms) of DEGs in U73122 (5µM) treated THP-1 cells compared to untreated cells. *P* value < 0.05 was considered statistically significant for KEGG enrichment analysis. (**C**) Functional enrichment bar chart (GO terms) of DEGs in U73122 (5µM) treated THP-1 cell compared to untreated cells. *P* value < 0.05 was considered statistically significant for GO enrichment analysis. (**D**) GSEA revealing significantly enriched cell cycle-related signaling pathways (NES > 1, adjusted *p* value < 0.05). (**E**) Heatmap showing the expression of representative cyclins

To determine whether PLCB1 inhibitor suppress the proliferation of leukemic cells, two cell lines (THP-1 and Molm-13) were treated with different concentrations of U73122 for 24 h and 48 h respectively. CCK-8 was used to detect cell proliferation. The results showed that U73122 inhibited cell proliferation in a dose-dependent manner (Fig. 5D; supplemental Fig. S5A). Furthermore, U73122 induced cell apoptosis (Fig. 5E; supplemental Fig. S5B). To explore the potential mechanism behind the induction of cell apoptosis following PLCB1 inhibition, transcriptome sequencing was performed on THP-1 cell line with and without U73122 treatment. The results showed that there were 674 up-regulated genes and 972 down-regulated genes (|LogFC|≥1, *p* value < 0.05) (Fig. 6A). Functional enrichment analysis of GO, KEGG and GSEA all showed that DEGs enriched in cell cycle-related signaling pathways (Fig. 6B-D). Next, we found the treatment of U73122 in THP-1 and Molm-13 cells resulted in an increase in the percentage of cells in the G1 phase and a decrease in S phase, indicating cell cycle G1/S arrest (Fig. 5F; supplemental Fig. S5C). Corresponding to that, western blotting analysis confirmed that the treatment of U73122 reduced the expression of CDK2 and cyclin E which play a key role in promoting G1/S transition [45] (Fig. 5G and Supplementary Fig. S5D). Additionally, transcriptome sequencing showed U73122 also reduced the other cyclins involved in G2/M phase transition such as CDK1 and cyclin B (Fig. 6E), which indicated the effect of U73122 on the cell cycle may be multifaceted and is not limited to G1/S phase transition.

## Discussion

MDSs are a group of clonal hematopoietic disorders and one of the typical presentations is myeloid cell dysplasia [46]. The underlying pathogenesis remains unclear. In this study, we sought to dissect the biological mechanisms that drive the occurrence and progression of MDS at the stem-cell level. We explored the developmental trajectories of HSPCs in MDS by pseudotime trajectory analysis using sc-RNA sequencing datasets. Obvious activation of immunity was observed in the early development stage of HSPCs in MDS patients when compared to HCs, especially the immune response against pathogenic microorganisms. Barreyro et al. [47] reported that chronic inflammatory diseases associated with activated innate immune signaling pathways often precede MDS, which was in line with our results. DEFA3, S100A8 and S100A9 were the top 3 up-regulated DEGs in module 1. Defensin alpha 3 (DEFA3) is a member of family of antimicrobial and cytotoxic peptides thought to be involved in host defense. The role of DEFA3 in the pathogenesis of MDS has not yet been reported. However, high levels of S100A8/A9 have all been implicated in pathogenesis of MDS/AML [48]. Schneider et al. [49] found the frequency of S100A8-expressing nucleated cells was substantially increased in bone marrow biopsies from subjects with del(5q) MDS as compared to those from normal individuals by immunofluorescence technology. Moreover, the frequency of S100A8 + cells in del(5q) MDS human bone marrow positively correlates with disease severity since it is functionally involved in the erythroid differentiation defect [49]. Additionally, the S100A8/A9 heterodimer, an endogenous TLR4 ligand, acts upstream of TNF-α and promotes the activation of the transcription factor NF-κB and the secretion of pro-inflammatory cytokines [50]. The increased signaling of S100A8/A9 mediated inflammasome formation leading to cell death, which may explain the apoptosis and cytopenia in early-stage MDS [48].

In the module 2 and module3, there are abnormalities in protein translation, protein localization and ribosome biogenesis, accompanied by the activation of a variety of inflammatory pathways, hematopoietic differentiation and apoptosis pathways. Aberrant cell growth and proliferation depend on increased protein synthesis and overactive translation which may induce abnormal ribosome biogenesis. Emerging evidence suggests that there is a close link between dysregulated ribosome biogenesis and tumorigenesis [51]. For example, heterozygous deletion of RPS14 has been linked to impaired erythropoiesis in the del(5q) MDS [49]. Liu et al. [18] found the expression of RPL31 and RPL21 mRNA in CD34 + cells of patients with low-risk -MDS, high-risk -MDS and sAML were significantly lower than those of healthy controls and positively correlated with the levels of hemoglobin and platelet. Although the mechanism of abnormal ribosome in the pathogenesis of MDS is still unclear and additional investigations are necessary to mechanistically understand the significance of ribosome biogenesis in MDS, these results indicate that ribosome-targeted therapy may be a promising approach for treating patients with MDS.

Another important feature of MDS is that it has an increased risk of evolution into AML. Here, we found that the proportion of CMP was the main cell cluster in the patients with EB. The results of cell cycle analysis indicated the CMP of MDS patients were in an active proliferative state. Thus, the proliferation of CMP may be an important event in the leukemic transformation. The cause of CMP amplification is not clear. Ganan-Gomez I et al. [52] reported that there were two MDS differentiation patterns, called CMP-pattern MDS and GMP-pattern MDS respectively. During blast progression, long-term (LT)-HSCs isolated from CMP-pattern MDS patients had significantly upregulated genes involved in promoting cell proliferation and survival (such as BCL2), while tumor necrosis factor (TNF)-induced nuclear factor-kappa B (NF-κB) signaling pathway were significantly upregulated in the lymphoid-primed multipotent progenitors (LMPPs) from GMP-pattern MDS patients. In our study, we also found the high expression of BCL2 along the pseudotime in MDS. Meanwhile, we observed MAML3 and PLCB1 that were associated with cell proliferation showed a more pronounced upward trend along the pseudotime. Moreover, these two genes were also in the top 20 up-regulated DEGs in the CMP subset compared to HCs. Through further validation, MALM3 and PLCB1 were found to be associated with EB, and the high expression of PLCB1 may be involved in rapid leukemic transformation. It is reported that PLCB1 can promote the proliferation of malignant cells by promoting G1/S phase transition [53]. Our results showed that PLCB1 inhibitor can suppress proliferation, induce cell cycle arrest, and activate apoptosis of leukemic cells in vitro. These results indicated that the high expression of PLCB1 might be one of the important factors for CMP proliferation in MDS, and thus plays an important role in the transformation of MDS into leukemia.

Some studies demonstrated that the mono-allelic deletion and promoter hypermethylation of PLCB1 are associated with the progression of high-risk MDS into AML [54, 55]. However, the relationship between the expression level of PLCB1 and leukemic transformation is controversial. Follo MY et al. [56]reported that a reduced expression of PLCB1 mRNA was observed in patients with high-risk MDS compared to healthy controls. But our data and public datasets both showed that the increased expression of PLCB1 were associated with EB and rapid leukemic transformation. The reason for this discrepancy may be that the samples they used are bone marrow and peripheral blood mononuclear cells while hematopoietic progenitor cells (CD34 + cells) were used in our study. In addition, the sample size may be another reason for the discrepancy since only a small sample was used in Follo MY’s study. Some studies revealed that PLCB1 is a positive regulator of myeloid differentiation and a negative regulator of erythroid differentiation and PLCB1 expression in MDS cells is usually increased during myeloid differentiation and is reduced during erythropoiesis [57–59]. On the other hand, PLCB1 can promote cell survival through inducing cell cycle progression in Friend erythroleukemia cells and pro-B-lymphoblastic cells [42, 53]. Therefore, we speculated that the abnormal high expression of PLCB1 may affect normal myeloid and erythroid hematopoiesis, and promote the proliferation of blast cells by regulating the cell cycle, thereby promoting the transformation of MDS to AML.

Notably, our study has certain limitations. Firstly, the single-cell transcriptomes of MDS patients and HCs come from two datasets which may cause data bias due to the batch effect. Secondly, some of these results are suggestive rather than conclusive since they are based on bioinformatic analysis of single-cell transcriptome and bulk transcriptome. Additionally, there is a lack of animal experiments to verify the role of PLCB1 in the transformation of MDS to AML.

In summary, we revealed the transcriptomic change of HSPCs in MDS patients along the pseudotime using trajectory analysis. In addition, we found PLCB1 plays an important role in the transformation of MDS into leukemia. For patients with high PLCB1 expression, pharmacologically targeting PLCB1 may be a potential treatment for halting MDS progression.

## Methods

### Patient samples

Bone marrow samples were obtained from 65 newly diagnosed patients with MDS. Of which, 40 cases were from Qingyuan People’s Hospital and 25 cases were from Nanfang Hospital. No treatment other than supportive care was given prior to sample collection. All biological samples were collected with informed consent according to procedures and conducted in accordance with the Helsinki Declaration, approved by the Ethics Committee of Qingyuan People’s Hospital (IRB-2023-104) and the Ethics Committee of Nanfang Hospital (Ethical approval No. NFEC-2020-304).

### Immunohistochemical analysis

The sections were incubated with anti-PLCB1 (#R30178, Zen-Bioscience, Chengdu, China; 1:200) at 4 °C overnight. After being washed with PBS, secondary antibody was added and was incubated for 1 h. Then the tissue was stained using DAB chromogenic solution. The labeling score of intensity was estimated as negative (0), weak (1), moderate (2) and strong (3). The extent of staining, defined as the percentage of positive stained cells, was scored as 0 (0%), 1 (≤ 10%), 2 (11–50%), 3 (51–80%) and 4 (> 80%). The total immunohistochemistry (IHC) score was obtained by multiplying the score of intensity and that of extent, ranking from 0 to 12.

### RT-qPCR

Bone marrow mononuclear cells (BMMNCs) from each sample were isolated by density gradient centrifugation using Ficoll gradients (Ficoll-Paque, TBD Science, Tianjin, China). After that, CD34 + hematopoietic stem and progenitor cells were sorted out from BMMNCs by immunomagnetic beads (Miltenyi Biotec, Bergisch Gladbach, Germany), Total RNA was extracted by TRIzol (Thermo Fisher Scientific, Waltham, MA, USA) and reverse transcribed to cDNA. Finally, qPCR was done. The qPCR amplification conditions: 40 cycles of 95 ℃ for 30 s, 95 ℃ for 5 s and 60 ℃ for 30 s, followed by a melting curve program, 65℃ for 60 s. The genes in our study have the same amplification conditions.

### Cell lines and cell culture

AML cell lines THP-1 and Molm-13 were obtained from the cell bank of Chinese Academy of Sciences (Shanghai, China), and were cultured in RPMI 1640 medium containing 10% or 20% FBS and 1% penicillin/streptomycin at 37 °C and 5% CO_2_.

### Cell viability assay

CCK-8 assay was used to detect cell viability. THP-1 cells or Molm-13 cells were seeded into 96-well plates at a density of 1 × 10^5^ cells/well with different concentrations of phospholipase C inhibitor (U73122) and cultured for 24–48 h. One hours before ending the incubation, 20 µl of CCK-8 was added to each well and incubated for 1 h. The total volume in each well was 200 µl. The absorbance was measured at 450 nm using a microplate reader (TECAN UK Ltd, UK).

### Cell-cycle analysis and cell apoptosis assay

THP-1 cells or Molm-13 cells in their logarithmic growth phases were added to a 6-well plate with 2 × 10^6^/well and incubated with different concentrations of U73122 for 24 h before cells were collected.

(1) The cells were fixed using 70% pre-cold ethanol overnight and then stained with 400 µl PI (50 µg/ml) and 100 µl RNase A (100 µg/ml) at room temperature for 15 min in the dark. Flow cytometry analysis was performed using Flow Cytometer (BD Biosciences) to determine the percentage of cells at every phase of the cell cycle.

(2) The cells were collected, washed twice using the binding buffer, and incubated with PE-labeled Annexin-V and 7-AAD (BD Biosciences) at room temperature in the dark for 25 min. Cell apoptosis was determined by flow cytometry.

### Western blotting

The following primary antibodies were used: anti-GAPDH (#2118, Cell Signaling Technology (CST), America; 1:1000), anti-cyclin D3 (#2936, CST; 1:1000), anti-CDK2 (#2546, CST; 1:1000), anti-CDK4 (#12,790, CST; 1:1000), anti-CDK6 (#13,331, CST; 1:1000), anti-cyclin E1 (#R24028, Zen-Bioscience, Chengdu, China; 1:1000) and anti-alpha tubulin (#11224-1-AP, Proteintech, Hubei, China; 1:10000). RIPA buffer containing protease and phosphatase inhibitors cocktail (New Cell&Molecular Biotech Co., Ltd, Suzhou, China) was used to extract cellular proteins. Used a BCA Protein Assay Kit (#PC0020, Solarbio, Beijing, China) measure protein concentrations. Equal amounts of protein were subjected to 10% SDS-PAGE before transferred to polyvinylidene fluoride (PVDF) membrane (Millipore, Billerica, USA). The specialized primary antibodies were used to incubate overnight under 4 °C conditions. The membranes were visualized with SuperECL Chemiluminescence detection reagents (Applygen Technologies Inc., Beijing, China).

### Single-cell RNA sequencing data processing

Raw files were processed with Cell Ranger 7.0.0 pipeline (10XGenomics, Pleasanton, CA, USA) using default mapping arguments. Reads were mapped to the human genome (GRCh38). Seurat R package (version 4.3) was used to analyze scRNA-seq data. We used the MergeSeurat function to merge datasets. The NormalizeData function was used to normalize the raw counts, and the FindVariableFeatures function was used to identify highly variable genes. Afterward, Harmony R package (version 0.1.1) was used to avoid the batch effect affecting downstream analysis [60]. The parameters of Harmony were used as followed: sigma = 0.1, tau = 0, block.size = 0.05, max.iter.harmony = 10, max.iter.cluster = 20, epsilon.cluster = 1e-05, epsilon.harmony = 1e-04. Cells with greater than 50% mitochondrial expression were removed from further analysis. PCA and UMAP were used to reduce the dimensions of the data, and the first two dimensions were used in the plots. Cell types were annotated based on the marker genes and their match to canonical markers (Cell cluster annotation was based on HSPC subsets specific marker genes reported previously). The R package SingleR (version 2.2.0), a novel computational method for unbiased cell type recognition of scRNA-seq, with reference transcriptomic dataset “HumanPrimaryCellAtlasData”, was utilized to infer the cell origin of each single cells independently and to identify cell types.

DEG analysis was performed using the function “FindMarkers”. We used Monocle 2(version 2.28.0) to construct the trajectory tree of all groups of cells, which indicated the distinct lineage differentiation potential for each group of cells. For identification of major patterns along the pseudotime, the top 1500 pseudotime-dependent genes were selected by the differentialGeneTest function with setting the parameters “fullModelFormulaStr” as “$\sim$sm.ns(Pseudotime)” and were clustered into three distinct patterns by k-means clustering [61]. BEAM analysis was used to get the expression patterns in branches during development [62]. GO, KEGG and GSEA analysis were preformed using the clusterProfiler R package (version 4.8.1). The terms with *p* values of less than 0.05 were considered as significant enrichments. We assigned a cell cycle phase (G1, S or G2/M) to each single cell using the ‘cyclone’ function in the Scran R package (version 1.28.1).

### Total RNA sequencing data processing

Total RNA of THP-1 cells which were treated by U73122 (5µM) or DMSO (0.1%) for 24 h (*n* = 3 each group) was extracted using TRIzol reagent (Thermo Fisher Scientific, Waltham, MA, USA). RNA sequencing was conducted on the Illumina HiSeq platform. The HISAT2 algorithm was used to map the sequenced reads to the human genome GRCh38 reference genome. DEG analysis (|LogFC|≥1, *p* value < 0.05) was performed using the limma R package (version 3.56.1). GO, KEGG and GSEA analysis were preformed using the clusterProfiler R package (version 4.8.1).

### Statistical analysis

Categorical variables were compared between the two groups using the chi-squared test, and continuous variables were compared using the Mann-Whitney U test. OS was calculated using the Kaplan–Meier method and compared by log-rank test. Statistical analyses were performed using R (version 4.3.0).

### Data availability

scRNA-seq data from 7 patients with MDS/sAML at diagnosis were obtained from Liu, Yumei et al. [18], which are deposited in the NCBI Sequence Read Archive under bioproject No. PRJNA 720,840. Another scRNA-seq data from 3 healthy controls came from Iskander, Deena et al. [19] (GSE156441). The mRNA expression profiles and relevant clinical information were downloaded from GSE114922 and GSE111085 datasets from the GEO database. The raw sequence data performed in this paper have been deposited in the Genome Sequence Archive (Genomics, Proteomics & Bioinformatics 2021) in National Genomics Data Center (Nucleic Acids Res 2022), China National Center for Bioinformation / Beijing Institute of Genomics, Chinese Academy of Sciences (GSA-Human: HRA009399) that are publicly accessible at https://ngdc.cncb.ac.cn/gsa-human. Other data generated or analyzed during this study are included in this manuscript. The code generated during this study is available from the corresponding author by reasonable request.

### Electronic supplementary material

Below is the link to the electronic supplementary material.

Supplementary Material 1
