# Supplementary material for: Single-cell transcriptomics dissects the transcriptome alterations of hematopoietic stem cells in myelodysplastic neoplasms
Source: J Transl Med. 2024 Apr 17;22:359. doi: 10.1186/s12967-024-05165-z (PMC11022353; doi:10.1186/s12967-024-05165-z)
Supplement: Supplementary file 1 — Supplementary Material 1 [file 12967_2024_5165_MOESM1_ESM.docx]

**Supplementary Materials for**

Single-cell transcriptomics dissects the transcriptome alterations of hematopoietic stem cells in myelodysplastic neoplasms

**This file includes:**

Supplemental Fig. S1 to Fig. S5

Supplemental Table S1

**Figure. S1.**


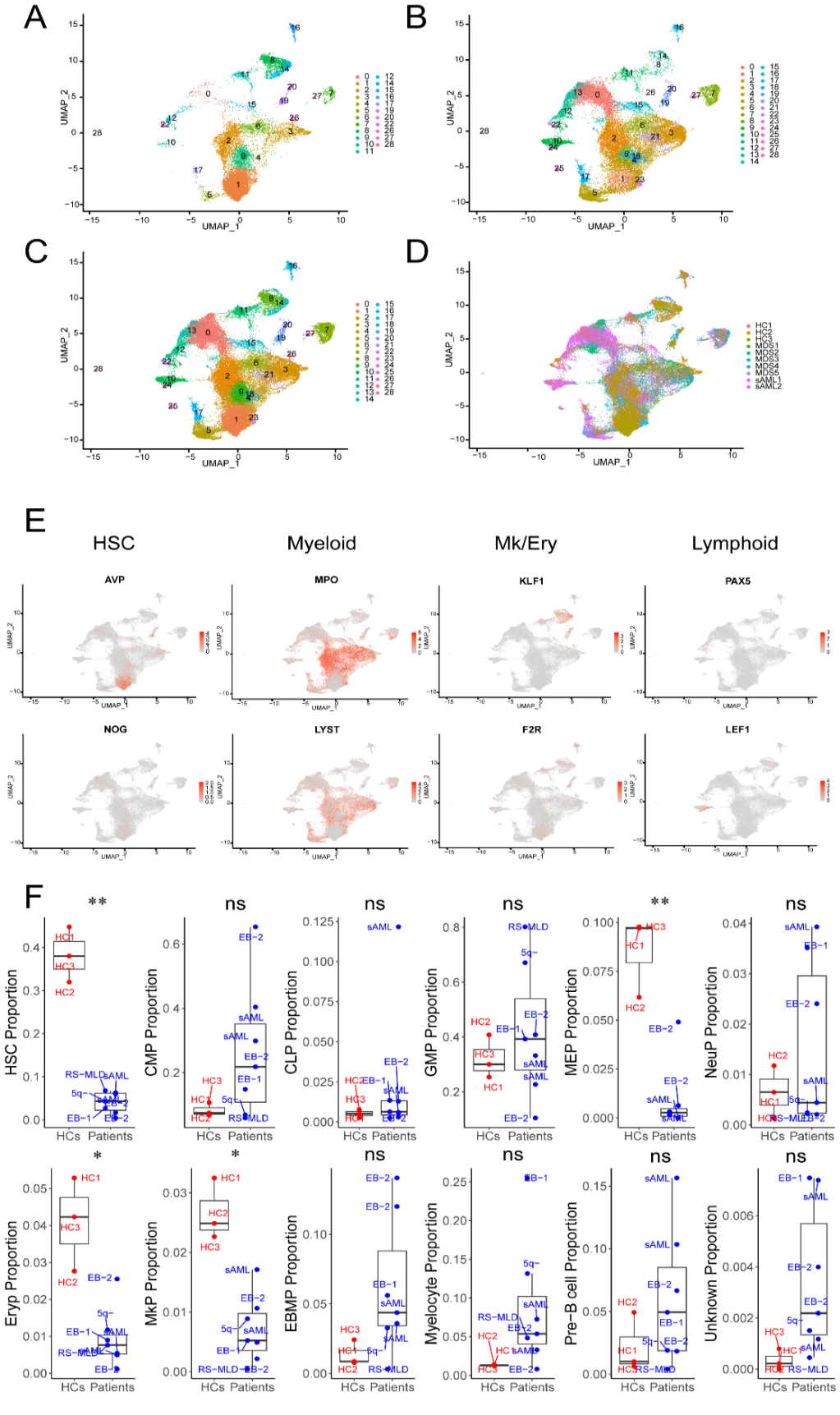


**Supplemental Fig. S1.** **Detailed UMAP of HSPCs from patients and HCs bone marrows, related to Fig. 1.** (A) The unsupervised clustering of 15434 cells in HCs. (B) The unsupervised clustering of 34914 cells in MDS/sAML. (C) The unsupervised clustering of total 50348 cells. (D) UMAP of HSPC subclusters. Cells are color-coded according to the samples. (E) UMAP plots displaying the expression of canonical marker genes during hematopoietic development. (F) Boxplot showing the fraction of each HSPC cluster in HCs (red) and MDS/sAML patients (blue) samples. The *p* values were calculated using two-tailed Student’s t test; **p* < 0.05，** *p* < 0.01.


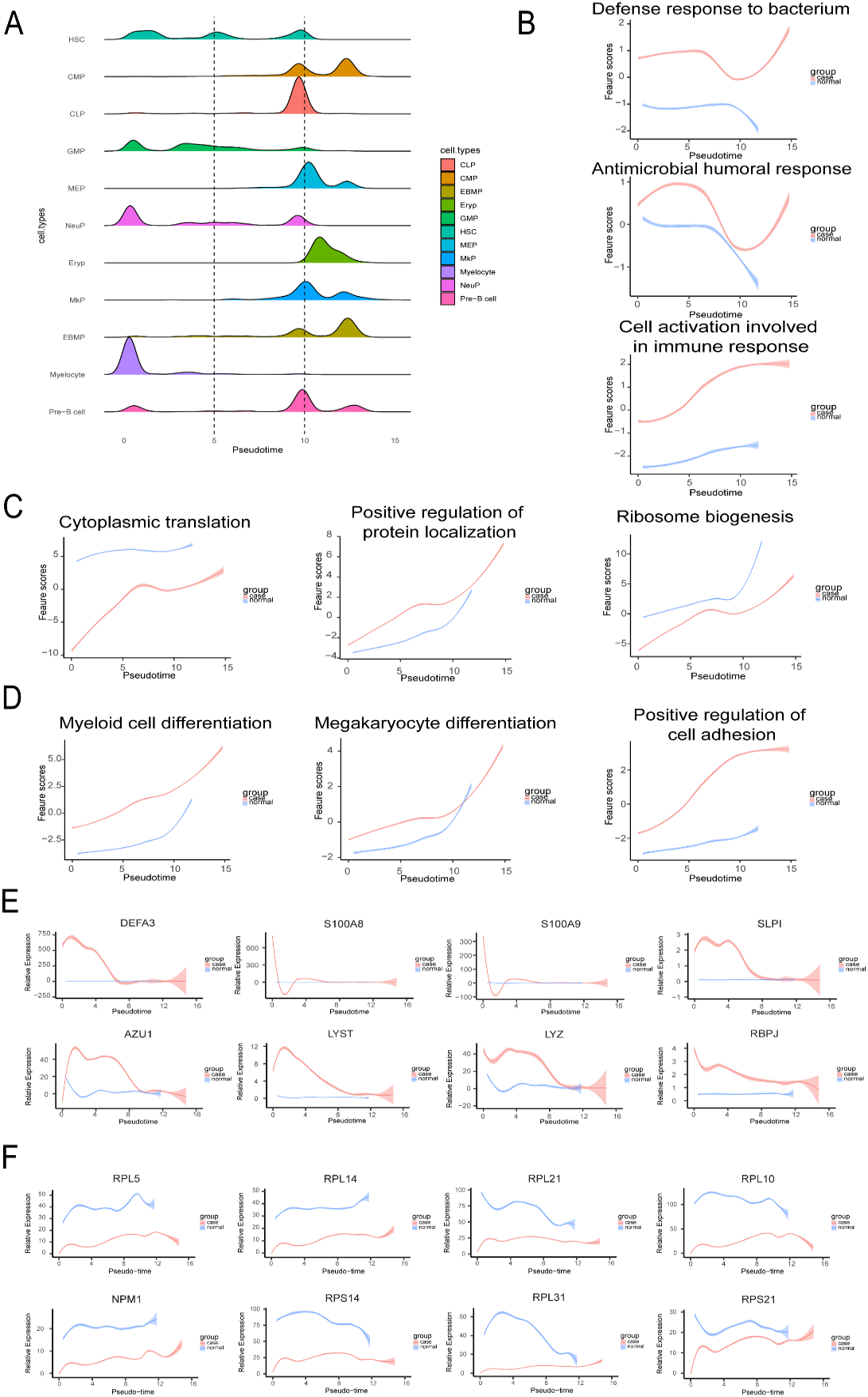
**Figure. S2.**

**Supplemental Fig. S2. Trajectory analysis of HSPC along the pseudotime, related to Fig. 2.** (A) The distribution of HSPC types along the pseudotime. (B) Two-dimensional plots showing the dynamic expression of scores for representative pathway in module 1 along the pseudotime in MDS/sAML (red) and HC (blue) groups. (C) Two-dimensional plots showing the dynamic expression of scores for representative pathway in module 2. (D) Two-dimensional plots showing the dynamic expression of scores for representative pathway in module 3. (E) Two-dimensional plots showing the dynamic expression of significantly enhanced genes in MDS/sAML patients compared with HCs along the pseudotime in module 1. |LogFC|≥1 and *p* value < 0.05 were used to define DEGs. (F) Two-dimensional plots showing the dynamic expression of significantly enhanced genes in MDS/sAML patients compared with HCs along the pseudotime in module 2. |LogFC|≥1 and *p* value < 0.05 were used to define DEGs.

Figure. S3.


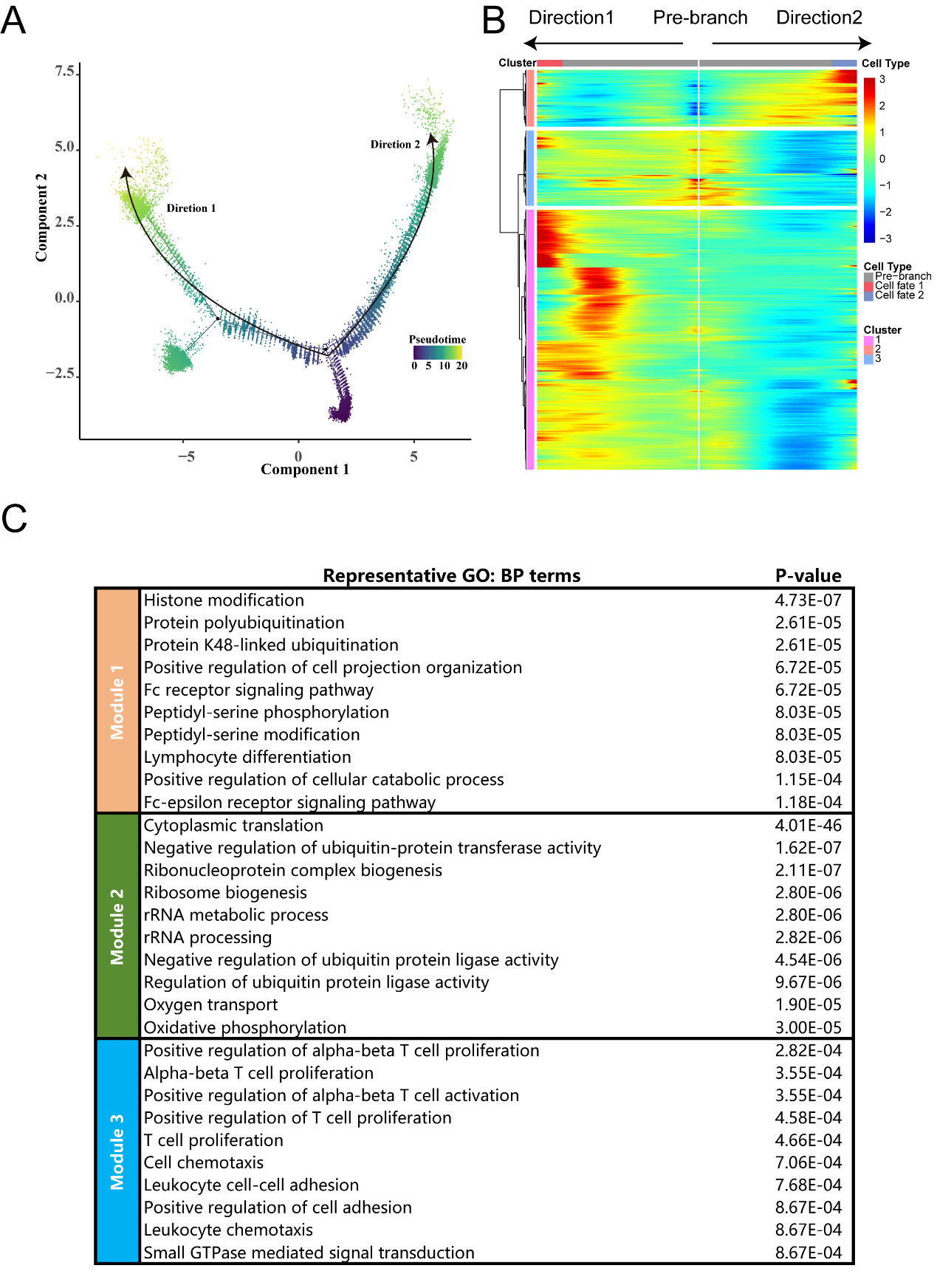


**Supplemental Fig. S3. Analysis of HSPC transition states in MDS and HC samples.** (A) Pseudotime-ordered analysis of HSPC and shows the direction of pseudotime. (B) Branched expression analysis modeling (BEAM) heat map depicting the expression of the top 1000 branch-dependent genes over pseudotime. |LogFC|≥1 and *p* value < 0.05 were used to define DEGs. Genes are clustered to three modules based on expression patterns across pseudotime. The branch point shown in the middle of heat map is the beginning of pseudotime. (C) Top 10 enriched GO: BP terms of each module. *P* value < 0.05 was considered statistically significant for GO enrichment analysis.

**Figure. S4.**


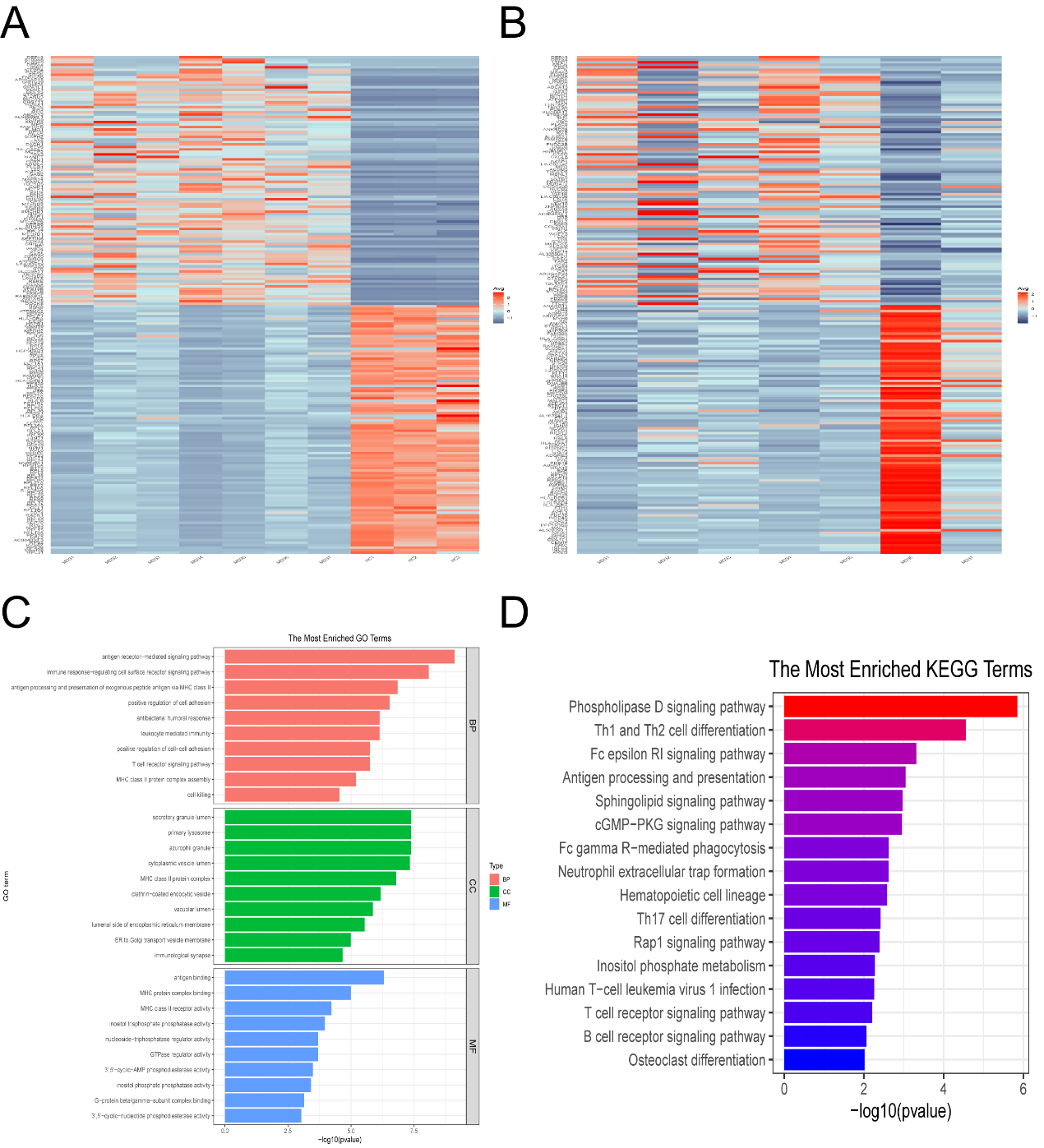


**Supplemental Fig. S4.** **Transcriptional changes of HSPCs in MDS.** (A) Heatmap of top 200 differentially expressed genes in total HSPCs of 7 MDS/sAML patients compared with 3 HCs. |LogFC|≥1 and *p* value < 0.05 were used to define DEGs. (B) Heatmap of top 200 differentially expressed genes in total HSPCs of 5 MDS patients compared with 2 sAML patients. |LogFC|≥1 and *p* value < 0.05 were used to define DEGs. (C) Functional enrichment bar chart (GO terms) of DEGs in total HSPCs of 5 MDS patients compared with 2 sAML patients. *P* value < 0.05 was considered statistically significant for GO enrichment analysis. (D) Functional enrichment bar chart (KEGG terms) of DEGs in total HSPCs of 5 MDS patients compared with 2 sAML patients. *P* value < 0.05 was considered statistically significant for KEGG enrichment analysis.

**Figure. S5.**


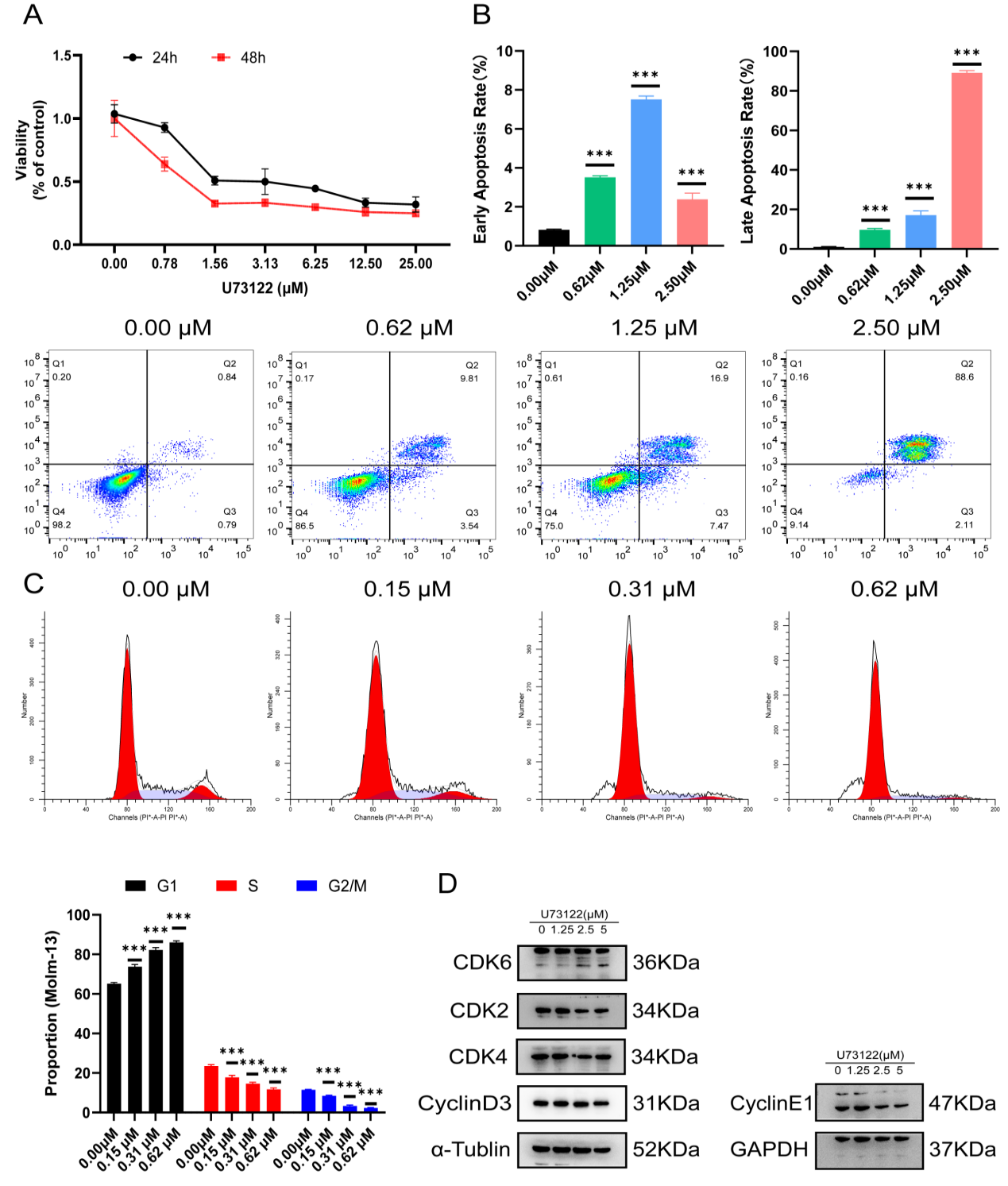


**Supplemental Fig. S5. PLCB1 inhibitor U73122 suppress proliferation, induce cell cycle arrest, and activate apoptosis of leukemic cells in vitro.** (A) CCK-8 assay of cell viability in Molm-13 cell line with different concentrations of U73122 for 24 and 48h respectively. (B) Cell apoptosis analysis of Molm-13 cells treated with 0.00μM, 0.62μM, 1.25μM or 2.5μM U73122 for 24h. (C) Cell cycle analysis of Molm-13 cells treated with 0.00μM, 0.15μM, 0.31μM or 0.62μM U73122 for 24h. (D) CDK6 and cyclin D3 expression stained unchanged while CDK2, CDK4 and cyclin E1 expression were down-regulated in Molm-13 cells after U73122 treatment.

**Table. S1. Patients’** **Demographic and Clinical Characteristics in our data.**

|  | Total (N=65) | PLCB1-Low (N=30) | PLCB1-High (N=35) | P |
| --- | --- | --- | --- | --- |
| **Age (years), median, range** | 59(24-91) | 58(25-77) | 60(24-91) | 0.56 |
| **Gender, no. (%)** |  |  |  | 0.17 |
| Male | 35(53.8%) | 20(62.5%) | 15(45.5%) |  |
| Female | 30(46.2%) | 12(37.5%) | 18(54.5%) |  |
| **Median WBC (range), ×10^9^/L** | 2.60(0.38-31.14) | 3.56(0.82-31.14) | 2.01(0.38-9.53 | 0.042 |
| **Median NE (range), ×10^9^/L** | 0.93(0.03-27.00) | 1.51(0.03-27.0) | 0.7(0.13-6.68) | 0.025 |
| **Median HGB (range), g/L** | 66(31-113) | 67(31-107) | 65(35-113) | 0.94 |
| **Median PLT (range), ×10^9^/L** | 68(9-307) | 73(9-289) | 65(11-307) | 0.90 |
| **MDS subtypes (WHO, 2022), no. (%)** |  |  |  | 0.008 |
| MDS with low blasts | 27(41.5%) | 19(59.4%) | 8(24.2%) | 0.004 |
| MDS, hypoplastic | 3(4.6%) | 2(6.3%) | 1(3.0%) | 1.00 |
| MDS with increased blasts (MDS-IB) | 35(53.8%) | 11(34.4%) | 24(72.7%) | 0.002 |
| MDS-IB1 | 7(20.0%) | 3(27.3%) | 7(29.2%) |  |
| MDS-IB2 | 28(80.0%) | 8(72.7%) | 17(70.8%) |  |
| **Cytogenetic** |  |  |  | 0.10 |
| Normal karyotype | 38(58.5%) | 22(68.8%) | 16(48.5%) |  |
| Aberrant karyotype | 27(41.5%) | 10(31.3%) | 17(51.5%) |  |
| **IPSS-R risk group, no. (%)** |  |  |  | 0.016 |
| Very low | 2(3.1%) | 0(0.0%) | 2(6.1%) | - |
| Low | 10(15.4%) | 8(25.0%) | 2(6.1%) | 0.044 |
| Intermediate | 16(24.6%) | 10(31.3%) | 6(18.2%) | 0.22 |
| High | 19(29.2%) | 10(31.3%) | 9(27.3%) | 0.72 |
| Very high | 18(27.7%) | 4(12.5%) | 14(42.4%) | 0.007 |
| **Leukemia transformation, no. (%)** | 14(21.5%) | 3(9.4%) | 11(33.3%) | 0.019 |

*WBC,* white blood cell count; *NE,* neutrophil; *HGB,* hemoglobin; *PLT,* platelet; *WHO*, World Health Organization; *IPSS-R*, international prognostic scoring system-revised.
